# Free Flap Outcomes of Microvascular Reconstruction after Repeated Segmental Mandibulectomy in Head and Neck Cancer Patients

**DOI:** 10.1038/s41598-019-44467-x

**Published:** 2019-05-28

**Authors:** Jennifer An-Jou Lin, Charles Yuen Yung Loh, Chia-Hsuan Tsai, Kai-Ping Chang, John Chung-Han Wu, Huang-Kai Kao

**Affiliations:** 1 Department of Plastic and Reconstructive Surgery, Chang Gung Memorial Hospital, Chang Gung University College of Medicine, Taoyuan, Taiwan; 2 St Andrew’s Centre for Plastic and Reconstructive Surgery, Broomfield Hospital, Court Road, Chelmsford, Essex, CM1 7ET United Kingdom; 3 Department of Plastic Surgery, Keelung Chang Gung Memorial Hospital, Keelung City, Taiwan; 4 Department of Otolaryngology-Head & Neck Surgery, Chang Gung Memorial Hospital, Chang Gung University College of Medicine, Taoyuan, Taiwan

**Keywords:** Head and neck cancer, Surgical oncology

## Abstract

This is the first study to investigate the impact of a second fibula flap or a soft tissue flap combined with bridging plate for a repeated segmental mandibulectomy reconstruction on flap outcomes in head and neck cancer patients. A retrospective comparative analysis (2007–2016) of 61 patients who underwent a second segmental mandibulectomy was performed. 20 patients underwent a fibula flap reconstruction whereas 41 had a soft tissue flap and plate reconstruction. No significant difference was seen in the operative time, total hospital stay, flap loss, re-exploration rates, plate exposure rate, or recipient site infection rate. On multivariate analysis, patients reconstructed with a soft tissue flap and bridging plate (odds ratio (OR) 3.997; 95% confidence interval (CI), 1.046–15.280, *p* = 0.043) and complications developed in previous surgery (OR 4.792; 95% CI, 1.120–20.493, *p* = 0.035) were shown to be independent predictors of a prolonged nasogastric tube dependence. The utilization of a soft tissue flap with plate is associated with comparative results of acute complication rate within 1 week, recipient site infection rate, and plate exposure rate to free fibula flaps alone. Free fibula flaps may result in a decreased risk for prolonged tube dependence compared to free soft tissue flap reconstructions.

## Introduction

With advances in operative techniques and perioperative care, the results of tumor ablation followed by immediate free tissue transfer for head and neck cancers have greatly improved. Nonetheless, the 5-year cumulative rate of recurrence or a second primary malignancy remains as high as 5% to 30% even after curative resection in patients with head and neck cancer^1–4^. For selected candidates, repeat resection is the first treatment of choice, and it provides acceptable safety and comparable long-term survival rates with primary resection. Complications due to previous surgery and/or radiotherapy such as osteoradionecrosis, fistula, and deformities can result in functional deficiency and decreased quality of life. In these scenarios, a sequential free tissue transfer is required. Among them, a sequential episode of microvascular reconstruction even a second osseous transfer is needed for patients who have a second mandibulectomy.

Reconstructive options for a segmental mandibular defect include vascularized bone grafts, bridging plates combined with soft tissue flaps, and non-vascularized bone grafts. The fibula flap is considered as the gold standard of choice owing to its length, bone stock, reliable pedicle, tolerance of dental implants, and low donor-site morbidity^3,5–7^. Acceptable results have also been reported using a soft-tissue flap and bridging plate in patients with poor disease prognosis or with large soft-tissue volume loss.

Several considerations exist before restoration of mandibular continuity after repeated mandibulectomy can be performed. These include a patient’s surgical fitness and disease status, defect location, involved structures, altered anatomic relationships, choice of recipient vessels, and other complications of the prior radiotherapy. The lack of recipient vessels and scarred tissue planes are all among the considerations that may increase the difficulty of the reconstruction after a second mandibulectomy. Although the free fibula flap provides rigid support, the soft tissue flap with bridging plate on the other hand, often is easier to perform as it provides ample soft tissue and easily achieves defect closure^2^.

To our knowledge, the current study provide the first evidence to compare free flap outcomes of a second fibula flap reconstruction or a soft tissue flap with bridging plate alone in patients undergoing a second repeated mandibulectomy. This study aims to fill that gap and facilitate preoperative patient counselling and clinical decision-making, and consequently affect the treatment strategies.

## Results

### Demographics

The overall study cohort comprised of 61 consecutive patients, with 20 patients reconstructed with the free fibula flap and 41 patients with soft-tissue flaps and bridging plates in the sequential mandibular reconstruction. Demographics and clinicopathological characteristics of the two groups are summarized in Table 1. The two groups were comparable with respect to gender predominance, age, body mass index (BMI), adjusted-Charlson Comorbidity Index (CCI), overall stage, defect location and type of free flap used in the first reconstruction, pre-operative radiation therapy (RT), and numbers of previous flap-related operations. The indication for repeated mandibular resection showed significant differences. All patients in the soft-tissue flap and plate group were of oncological purpose (100% *vs*. 65%, *p* < 0.001), whereas a higher proportion in free fibula flap group was due to osteoradionecrosis (35% vs. 0%, *p* < 0.001). The free fibula flap group has a significantly longer interval between the first and second mandibular reconstruction (3.88 ± 1.89 years *vs*. 2.71 ± 1.82 years, *p* = 0.017). Lastly, overall complications developed after previous reconstruction are significantly lower in the free fibula flap group (50% *vs*. 80.5%, *p* = 0.019).

**Table 1:** Baseline characteristics.

	Fibula *n* (%)	Soft tissue + Plate *n* (%)	*p*
Number	20	41	
Sex (M/F)	19 (95.0)	41 (100.0)	0.328
Age (yr) (Mean ± SD)	56.42 ± 11.88	55.80 ± 7.52	0.854
BMI (Mean ± SD)	21.99 ± 3.05	23.03 ± 4.41	0.534
CCI score (Mean ± SD)	3.60 ± 1.19	3.59 ± 1.22	0.949
Reconstruction indication			<0.001
Tumor (2^nd^ primary/recurrence)	12/1 (65.0)	36/5 (100.0)	
ORN	7 (35.0)	0 (0.0)	
Overall stage			0.722
I/II	4 (20.0)	10 (24.4)	
III/IV	9 (45.0)	31 (75.6)	
Shaw defect classification in 1st surgery			0.934
I	7 (35.0)	12 (29.3)	
II	10 (50.0)	17 (41.5)	
III	1 (5.0)	3 (7.3)	
IV	1 (5.0)	4 (9.8)	
Missing	1 (5.0)	5 (12.2)	
Reconstruction in 1st surgery			
Fibula	12 (60.0)	23 (56.1)	
Soft tissue + plate/double flap	8 (40.0)	18 (43.9)	
Interval between 1st and 2nd op (years) (Mean ± SD)	3.88 ± 1.89	2.71 ± 1.82	0.017
Complications in 1st surgery			
Flap-related complications <1 wk	1 (5.0)	8 (19.5)	0.249
SSI	6 (30.0)	22 (53.7)	0.105
Total flap loss	1 (5.0)	8 (19.5)	0.249
Partial flap loss	3 (15.0)	9 (22.0)	0.734
Plate exposure	4 (20.0)	17(41.5)	0.151
Other late sequelae	4 (20.0)	11 (26.8)	0.754
Overall	10 (50.0)	33 (80.5)	0.019
Radiation			
Pre-operative RT	18 (90.0)	40 (97.6)	0.248
Post-operative RT	7 (35.0)	23 (54.8)	0.174
flap-related op before 1st op (Mean ± SD)	0.80 ± 1.24	0.73 ± 1.30	0.451
flap-related op between 1st and 2nd op (Mean ± SD)	0.85 ± 1.27	1.17 ± 1.55	0.439

BMI, body mass index; CCI, Charlson comorbidity index; ORN, osteoradionecrosis; SSI, surgical site infection (recipient); RT, radiation therapy.

### Operative variables

Operative characteristics of the two groups are summarized in Table 2. There were no statistically significant differences noted between the two groups in variables including the defect classification, defect size, pre-operative hemoglobin, pre-operative albumin, estimated blood loss (EBL), frequency of intra-operative blood transfusions, operative duration, ischemia time and location of recipient vessels. Free fibula flap has a significantly longer ischemic time (209.68 ± 57.96 vs. 117.55 ± 44.44, *p* < 0.001).

**Table 2:** Operative variables.

	Fibula *n* (%)	Soft tissue + Plate *n* (%)	*p*
Numbers	20	41	
Defect type by Shaw classification			0.357
I	4 (20.0)	2 (4.9)	
II	6 (30.0)	12 (29.3)	
III	3 (15.0)	1 (2.4)	
IV	7 (35.0)	9 (22.0)	
Missing	0 (0.0)	17 (41.5)	
Defect size (cm^2^)			
Skin (Mean ± SD)	74.3 ± 36.7	74.4 ± 40.0	0.99
Mucosa (Mean ± SD)	53.3 ± 36.6	46.0 ± 25.4	0.37
Bone (Mean ± SD)	7.3 ± 3.6	8.1 ± 2.9	0.39
Blood loss (mL) (Mean ± SD)	293.50 ± 180.36	380.24 ± 319.19	0.568
Blood transfusion	10 (50.0)	15 (36.6)	0.408
Op time (min) (Mean ± SD)	676.84 ± 175.63	599.05 ± 131.85	0.19
Ischemic time (min) (Mean ± SD)	209.68 ± 57.96	117.55 ± 44.44	<0.001
Location of recipient vessels			1
Ipsilateral neck	5 (25)	10 (24.4)	
Contralateral	13 (65)	28 (68.3)	
Outside neck	2 (10)	3 (7.3)	

Hb, hemoglobin; Alb, albumin.

### Clinical outcomes

Morbidity with respect to length of stay (intensive care unit (ICU)/Hospital), acute flap complications, re-exploration rates, flap loss (total/partial), surgical site infection (SSI), plate exposure and other late complication was not significantly different between the two groups (Table 3). Long-term nasogastric tube dependency was 61% in the soft-tissue and plate group, which was significantly higher than in the free fibula flap group (20%) (*p* = 0.006).

**Table 3:** Clinical outcomes.

	Fibula *n* (%)	Soft tissue + Plate *n* (%)	*p*
Number	20	41	
ICU stay (days) (Mean ± SD)	8.70 ± 6.01	8.15 ± 4.84	0.4
Hospital stays (days) (Mean ± SD)	29.50 ± 17.06	29.32 ± 12.83	0.678
In-hospital mortality	0 (0.0)	1 (2.4)	1
Acute recipient site complication within 1 wk			0.739
arterial related	0 (0.0)	1 (2.4)	
venous related	1 (5.0)	3 (7.3)	
venous + arterial related	0 (0.0)	2 (4.9)	
neck hematoma	2 (10.0)	1 (2.4)	
Re-exploration rate	3 (15.0)	7 (17.1)	1
Partial flap loss	4 (20.0)	12 (29.3)	0.544
Total flap loss	3 (15.0)	7 (17.1)	1
Recipient site infection	10 (50.0)	23 (56.1)	0.786
Plate exposure	6 (30.0)	12 (29.3)	1
Other late sequelae	10 (50.0)	16 (39.0)	0.582
Feeding			0.006
Tube dependent	4 (20.0)	25 (61.0)	
Regular	16 (80.0)	16 (39.0)	

ICU, intensive care unit.

### Risk factors of four major complications

**Figure 1:**
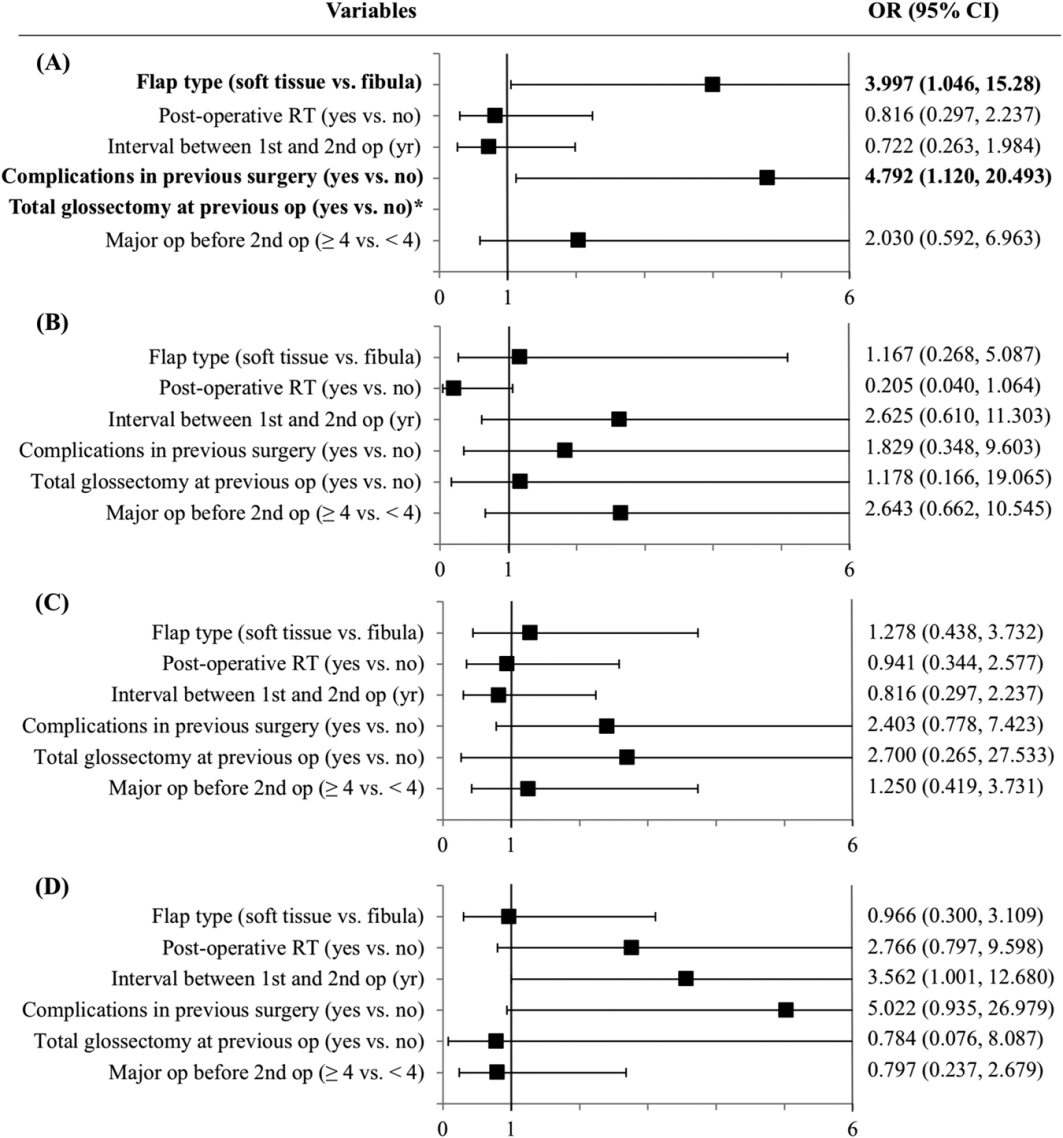
Forest plot showing the adjusted predictors for (**A**) long-term tube dependency (**B**) acute complications within 7 days (**C**) recipient site infection (**D**) plate exposure. Bold text indicates statistical significance (p < 0.05). *Indicates that all the patients underwent total glossectomy remained tube dependent and was not included in the multivariable analysis.

In the adjusted multivariable logistic regression analysis (Fig. 1), soft tissue flap and plate [OR = 3.997 (95% confidence interval (CI), 1.046–15.280), *p* = 0.043], complications developed in previous operation [OR = 4.792 (CI, 1.120–20.493), *p* = 0.035], and total glossectomy (*p* = 0.039) were three independent predictors for long-term tube dependency. No risk factor associations were identified for acute complications, surgical site infection (SSI), or plate exposure.

## Discussion

In this study, we examined the feasibility and outcomes of using the second fibula flap or a soft tissue flap with bridging plate for reconstruction of patients who have undergone repeated segmental mandibulectomy in head and neck cancer. To the best of our knowledge, this is the first comparative study and is the largest case series comprising of longitudinal and sequential observations of surgical outcomes over a 10-year period in a total of 61 patients receiving sequential free flap reconstruction after repeated segmental mandibulectomy. Our study shows that a second free fibula flap reconstruction after a repeated segmental mandibular resection has comparable results with the soft-tissue flap and bridging plate in terms of the operative time taken between each flap type, intensive care unit stay, total hospital stay, acute complications, flap loss, re-exploration rates, recipient site infection rate, or plate exposure rate. However, we found that in patients reconstructed with a soft tissue flap combined with bridging plate, complications developed in previous surgery and total tongue resection are three independent predictors of a prolonged nasogastric tube dependence postoperatively.

Although the concept of a sequential fibula flap reconstruction for repeated mandibulectomy has been reported before, prior studies have been limited to small sample sizes or were of a descriptive nature. In contrast, few studies addressed the role of soft-tissue flaps in these patients with difficult repeat reconstructions and no further investigations were performed.

A major obstacle in performing microvascular reconstruction after repeated segmental mandibulectomy is the technical complexity of the procedure. Direct comparison between different flaps is fraught with challenges, particularly because different flaps are often selected based on the defect, patients’ related factors, and prognosis. The criteria used to select patients for a second fibular flap or a soft tissue flap combined with a plate varies among surgeons. However, all the reconstructive microsurgeons were present on staff throughout the entire study period and were equally distributed in case volume, indicating that the impact of surgeons’ variation on flap outcomes is minimal. The Shaw classification, which can be easily interpreted based on patients’ image, provides a guidance in classifying mandibular defects^8^. Of note, no significant difference was found in defect locations, defect size, bone defect, location of recipient vessels, and pre-existing disease between these two groups and thus the influence of bias could be weakened and veiled to some extent in the current study.

Tube feeding may be beneficial to head and neck cancer patients, both pre and post treatment, in order to provide temporary nutritional support. However, poor restoration of swallowing function and persistent dysphagia would lead to dependence on feeding tube, especially in elderly patients, an advanced tumor stage, or in patients requiring aggressive radiotherapy. There is no consensus on the definition of tube dependency. In published papers, a broader definition for long-term tube use was more than 6 weeks and less than 12 months after the completion of treatment^9^. The current study defined long-term tube dependency as the use of a tube feeding for more than one year after the reconstruction. All the patients in our series had nasogastric tube insertion. No esophagostomy nor gastrostomy were performed. Of note, the pre-operative intake status were recorded and there were no patients using a nasogastric tube prior to the sequential mandibulectomy and free flap reconstruction.

Risk factors associated with prolonged tube dependency in patients with head and neck cancer include patient’s characteristics, tumor stage/location and medical/surgical treatment related factors^10–12^. Patients’ pre-operative condition such as old age, weight loss, and tabacco use have been identified as independent factors of tube dependency in previous studies^10,11^. Acute side effects of radiotherapy may result in mucositis and xerostomia. Mucositis was observed by Manikantan *et al*. in almost 100% of patients who underwent chemoradiation, and 40% in patients receiving chemotherapy alone^12^.

In the current study, three surgical treatment-related risk factors for long-term tube dependence were identified in this study: reconstruction with a soft tissue flap and plate, previous total glossectomy, and complications that developed from previous surgery. Repeated resection may lead to a larger defect and larger loss of bony support. Moreover, all the patients in our series had received radiotherapy pre-operatively. This was a major cause of progressive scar contracture and tissue fibrosis. A second fibula flap reconstruction is still advantageous in re-establishing mandible continuity, with 80% of patients achieving regular oral intake within one year post-operatively. On the contrary, 61% of patients reconstructed with a soft tissue flap and bridging plate remained on NG-tube feeding. Mericli *et al*. also demonstrated comparative results of tube feeding after the second fibula flap reconstruction when compared to the first episode^3^. Higher rates of tube dependence after soft-tissue flap reconstruction were also reported in the repeated reconstruction for head and neck cancer^13^. We believe that the osseous support is the main contributing factor to restoration of swallowing functions. Also, a bulky soft-tissue flap tends to act as an adynamic segment disturbing swallowing efficiency. The fibula flap on the other hand has inherent connections between the bone and soft tissue components which allow dynamic movement together when performing swallowing functions. A soft tissue and plate reconstruction are two separate units and over time, the effect of gravity will preferentially create a drag on the soft tissue component which will result in the patient being unable to effectively chew or close their mouth. Several previous studies demonstrated that resection of intraoral soft tissue, especially when the defect involves the tongue or the mouth floor also contributes to poor oral function^2,3^. When a more extensive glossectomy is combined with mandible resection, recovery of functional swallowing is poorer because patients cannot adequately mobilize the tongue and elevate the larynx, which impacts cricopharyngeal opening. Furthermore, when patients experienced more complications in previous surgery, an increased insult to soft tissues will increase levels of inflammation locally which in turn results in an altered anatomy with more severe scar contracture and fibrosis. This scar and fibrosis will also contribute to impaired oral intake function.

The flap success rate of 85% in the fibula group and 82.9% in the soft tissue and plate group was acceptable and showed no difference. Some might concerned about the donor-site morbidity after bilateral fibula flaps were harvested. However, Lin *et al*. has conducted an a study specifically addressing this issue and found that the fibula flap has a minimal and acceptable functional deficit^14,15^. Similarly, all patients in our series freely ambulated post-operatively and there were no gait imbalances or other functional deficits noted clinically.

Although our database enabled us to stratify our analysis such that we were able to identify the independent predictors, the present study has some limitations, including its retrospective design and that it was a single-center study. Bias exists in many forms for patients treated at different time-points across a 10-year period. The insufficient sample size was due to the rarity of the second segmental mandibulectomy. To overcome these limitations, a multi-institutional prospective study would be ideal, although many methodological challenges still would exist.

In conclusion, free fibula flap reconstruction in second mandibular resection, relative to soft-tissue flap and bridging plate, showed comparable in-patient mortality and medical complications. However, the free fibula flap is associated with lower risk of prolonged tube dependency. These results offer important insights into the comparative effectiveness of these reconstructive approaches and can provide guidance on preoperative counselling with regards to patients’ long-term functional outcomes and quality of life.

## Patients and Methods

This study was approved by the Institutional Review Board at Chang Gung Memorial Hospital, Taiwan (IRB Number: 201800682B0). Informed consent was obtained from all patients and the investigation was performed in accordance with the approved guidelines. A total of 61 consecutive patients with head and neck cancer who had undergone a second occasion of segmental mandibulectomy followed by microsurgical free flap reconstruction at Chang Gung Memorial Hospital, Linkou Medical Center, Taiwan between Jan. 2007 and Dec. 2016 were retrospectively reviewed. The indications for the repeated mandibular resection included cancer recurrence, second primary cancer, or osteoradionecrosis. Patients who underwent free flap reconstruction as a salvage procedure for failed initial free flaps were excluded. Other exclusion criteria were a follow-up of less than 12 months and those with incomplete medical records.

Patient-level data included gender, age, body mass index (BMI), preoperative serum albumin, recurrence status, overall stage, interval between two surgeries (in years), history of radiation therapy (RT), and other flap-related operations. Pre-existing co-morbidities were assessed and quantified according to the age adjusted-Charlson Comorbidity Index (CCI) scores^16^. The mandibular defect, type of free flap used, and complications from the first operation were also recorded.

The operative records were reviewed to determine the operative time, estimated blood loss (EBL), at least one intra-operative blood transfusion, and ischemia time. The Shaw classification was used to classify the location and the relative complexity of the oromandibular defect^8^. A titanium mandibular plate fixation system (Synthes, Inc., Zuchwil, Switzerland) was used to span the mandibular defect or secure the fibula flap in all cases. The laterality of recipient vessels used in the second surgery was defined relative to the first defect.

Our primary outcomes of interest were the following: microvascular flap-related complications (arterial or venous thrombosis, and recipient site hematoma) within seven days of surgery^17^, flap loss (partial/total), flap re-exploration, post-operative surgical site infections (SSI), plate exposure, other long-term complications, and feeding conditions postoperatively. SSI is defined by clinical identification of erythema, purulent discharge or wound dehiscence at the recipient site within the first 30 days after surgery^18^. The use of a nasogastric tube, esophagostomy or gastrostomy for more than 12 months after the second reconstruction was defined as long-term tube-dependent. Secondary outcomes included in-hospital mortality and length of stay (intensive care unit (ICU) and hospital).

### Statistical analysis

Continuous data were expressed as means and standard deviations whereas categorical data were presented as frequencies and percentages. Group comparisons were performed using Fisher’s exact test and Mann-Whitney U test for categorical variables and continuous variables respectively. Multivariable binomial logistic regression analysis was conducted to determine whether certain co-variates were associated with the occurrence of four outcome events: acute complications, wound infection, plate exposure, and tube dependency. Analyses were performed using SPSS Statistics software version 22. All *p* values were two-tailed, and values lower than 0.05 were set for statistical significance.

## Data Availability

The datasets generated and analysed during the current study are available from the cor-responding author on reasonable request.
